# FGA Controls VEGFA Secretion to Promote Angiogenesis by Activating the VEGFR2-FAK Signalling Pathway

**DOI:** 10.3389/fendo.2022.791860

**Published:** 2022-04-13

**Authors:** Hui Li, E. Cai, Hongyan Cheng, Xue Ye, Ruiqiong Ma, Honglan Zhu, Xiaohong Chang

**Affiliations:** ^1^ Department of Obstetrics and Gynaecology, Peking University People’s Hospital, Beijing, China; ^2^ Center of Gynaecological Oncology, Peking University People’s Hospital, Beijing, China

**Keywords:** endometriosis, fibrinogen alpha chain (FGA), angiogenesis, VEGFA-VEFGR2 pathway, endothelial cells

## Abstract

**Background:**

Our previous work revealed the high expression of fibrinogen alpha chain (FGA) in patients with endometriosis (EM) and that it could promote the migration and invasion of endometrial stromal cells. Angiogenesis is the key condition for the development of EM. This study was aimed to elucidate the role of FGA in endometrial stromal cells involved in angiogenesis in EM.

**Methods:**

Immunohistochemistry was used to detect the microvessel density (MVD) and VEGF expression in the eutopic endometrium samples from EM and non-EM. The conditioned medium (CM) of human primary eutopic endometrial stromal cells (EuESC) and immortalized endometrial stromal cell line hEM15A with FGA knockdown were collected and used to treat human umbilical vein endothelial cells (HUVECs). Then, tube formation assay, EdU assay, wound assay, transwell assay and flow cytometry assays were performed to assess the function of HUEVCs *in vitro*. The angiogenic capability of HUVECs was further measured using a matrigel plug assay with BALB/c nude mice *in vivo*. Immunofluorescence was used to detect the expression of F-actin and VE-cadherin. RT-PCR and western blotting were used to detect the expression of angiogenesis-related factors in endometrial stromal cells and downstream signalling pathways in HUVECs.

**Results:**

MVD and VEGF expression in the eutopic endometrium of EM patients were significantly higher than those in the normal endometrium of non-EM patients, and the increased MVD in EM indicates an increased risk of recurrence. Functionally, we found that CM of endometrial stromal cells with FGA knockdown could inhibit HUEVCs migration and tube formation *in vitro* and *in vivo*, while having no significant effect on HUVECs proliferation, apoptosis and cell cycle. Mechanically, the expression of VEGFA, PDGF, FGF-B, VEGF, MMP-2 and MMP-9 was reduced in hEM15A cells with FGA knockdown. CM of hEM15A cells with FGA knockdown reduced the number of microfilaments and pseudopodia, as well as the expression of VE-cadherin, and inhibited the activity of VEGFR2 and the FAK signalling pathway in HUVECs.

**Conclusion:**

Our study demonstrated FGA could enhance the interaction between endometrial stromal cells and HUVECs *via* the potential VEGA-VEGFR-FAK signalling axis and promote EM angiogenesis, revealing a promising therapeutic approach for EM.

## Introduction

Endometriosis (EM) affects approximately 10% of women of reproductive age and is a common gynaecological disorder characterized by the presence of endometrial cells outside the uterus (1, 2). It causes pelvic pain in 30%-50% of patients and infertility in 50% of patients, which substantially impairs quality of life (3, 4). Although various theories have been proposed, the aetiology and causative mechanisms of EM remain poorly understood (5). One of the most accepted theories is the retrograde menstruation theory, which indicates that endometrial tissue fragments flow from the uterus into the pelvic cavity through the fallopian tube during the menstrual cycle. There are several steps needed for endometriotic lesions to survive and grow in the pelvic cavity, including the adherence of endometrial cells/tissues to the peritoneum, the invasion of these cells and tissues and the establishment of a stable blood supply (6). Adequate oxygen and nutrition are necessary for the survival of endometriotic lesions. Thus, the establishment of new blood vessels is essential for the implantation and maintenance of shed endometrial tissue (7).

Fibrinogen is a 340-kDa glycoprotein and consists of three pairs of nonidentical polypeptide chains, termed α-, β-, and γ-chains, which are involved in blood clot formation (8). As a subunit of fibrinogen, the fibrinogen α chain (FGA) contains 866 amino acids and has a molecular weight of approximately 95 kDa. Several studies have demonstrated that elevated levels of FGA are closely related to the progression and metastasis of tumours and could be potential biomarkers for many cancers, including multiple myeloma (9), colorectal cancer (10), gastric cancer (11), and breast cancer (12). However, only our two studies have closely examined the role of FGA in the development of EM. Our previous study found increased expression of FGA in the serum and eutopic and ectopic endometrial tissues of women with EM. Moreover, suppression of FGA significantly inhibited the migration and invasion abilities of the endometrial stromal cell line hEM15A, which indicated that FGA may play an important role in the development of EM (13, 14). Based on these findings, we explored the roles of FGA in the biological behaviours of human primary EuESCs and the underlying mechanisms involved. Consistent with a previous report on hEM15A, FGA downregulation significantly inhibited the motility of EuESCs. Furthermore, we found that FGA plays a crucial role in the pathogenesis of EM by promoting cell migration and invasion through Arg-Gly-Asp (RGD) sequences binding integrin αvβ3 and activating the FAK/AKT/MMP-2 signalling pathway (15). Previous studies clearly show that FGA plays vital roles in EM progression and that its roles in EM development may be dependent on cellular migration and invasion. However, the role of FGA in EM-related angiogenesis has not yet been determined.

Angiogenesis, as one key process in the pathogenesis of EM, is a complex, multistep continuous process involving numerous factors (16). The specific factors are as follows: the release of angiogenic factors, the proliferation and migration of endothelial cells (ECs), and the degradation of the basement membrane and extracellular matrix (ECM), which finally form blood capillaries and the vascular network (17). Fibrinogen is an ECM protein involved in blood clot formation but also plays roles in tumour angiogenesis and metastasis. Early studies revealed that fibrinogen could modulate various physiological and pathophysiological events, such as angiogenesis and wound healing, by interacting with vascular ECs (18, 19). Cheng et al. found that fibrinogen upregulation increased the number of vascular ECs and other endogenous cells and promoted collagen deposition, directly resulting in new blood vessel formation (20). Previous studies also indicated that fibrinogen could associate with vascular ECs to promote angiogenesis through the binding of RGD residue of FGA to specific integrins (21, 22). Although potentially relevant, the role of FGA in the physiology of angiogenesis is unclear.

In this study, we aimed to evaluate the angiogenic activity in women with EM and then explore the effect and underlying mechanism of FGA in endometrial stromal cells on angiogenesis *in vitro* and *in vivo*.

## Materials and Methods

### Ethical Approval and Consent to Participate

The study was conducted in accordance with the principles of the Declaration of Helsinki and was approved by the Ethics Committee of Peking University People’s Hospital (No. 2020PHB161-01). Informed written consents were obtained from all patients prior to their enrollment in this study. All procedures involving animals were carried out according to the European Community Council Directive of 24 November 1986, and ethical approval was granted by the Animal Experiment Ethical Committee of Peking University People’s Hospital (No. 2021PHE027).

### Patients Information and Tissue Samples

Eutopic endometrium specimens were obtained from 31 patients with EM undergoing hysteroscopy combined with laparoscopy treatment at Peking University People’s Hospital from August 2017 to August 2019. As controls, normal endometrial specimens were collected from 40 women without EM by dilatation and curettage during the same period. The basic information of patients was seen in **Table 1**. No participants were pregnant, had menopause or history of cancer, and none had used steroid hormones within 3 months prior to study enrollment, nor did they have acute inflammatory disease or infection or systemic autoimmune disease.

**Table 1:** Clinical data of patients with endometriosis and without endometriosis (control).

Clinical Characteristics	Control (n=40)	Endometriosis (n=31)	*P*
Age (mean±SEM)	33.50±0.77	31.77±0.69	0.110
Menstrual cycle			
Proliferative phase	19	24	0.025
Secretory phase	21	7	
Dysmenorrhea			
Yes	11	22	<0.001
No	29	9	
Infertility			
Yes	22	27	0.004
No	18	4	
MVD	10.85±0.40	14.68±0.63	<0.0001

### Source and Culture Conditions of Cells

hEM15A is an immortalized stromal cell line derived from eutopic endometrial tissue of EM patients, which is preserved in the China Center for Type Culture Collection and Cell Resource Center of Peking Union Medical (23). Detailed performance about isolation and identification of human primary eutopic endometrial stromal cells (EuESCs) from endometriosis patients were described in our previous study (15). The HUVECs was a generous gift from Orthopedic Oncology Laboratory of Peking University People’s Hospital. EuESCs and hEM15A cells were cultured in Dulbecco’s modified Eagle’s medium (DMEM)/F12 (Gibco, USA) supplemented with 15% fetal bovine serum (FBS, Gibco) and 1% penicillin/streptomycin. HUVECs were cultured in DMEM/F12 (Gibco, USA) supplemented with 10% FBS (Gibco) and 1% penicillin/streptomycin. All cells were maintained at 37°C in a humidified atmosphere with 5% CO2 atmosphere.

### Lentiviral Infection and Preparation of Conditioned Medium

Lentiviral vectors containing shNC (negative control, NC) and shFGA were purchased from Hanheng Biotechnology (Hanheng Biotechnology Co., Ltd., Shanghai, China). The viral supernatant was used to transduce hEM15A cells and primary EuESCs according to the manufacturer’s protocol and selected with 4 µg/ml puromycin for about 2 weeks. Cells were maintained in DMEM/F12 containing 15% FBS and then transfection efficiency was verified by RT-PCR and western blotting. As for conditioned medium preparation, shFGA cells and shNC cells were respectively seeded at a density of 3×10^6^ cells in 75 mm culture flakes containing DMEM/F12 supplemented with 15% FBS. When cells reached about 80% confluence, rinsed with PBS twice and then cultured for an additional 48 hours in DMEM/F12 with 5% FBS. Subsequently, the culture medium was collected and centrifuged at 2000 g for 20 min. The conditioned medium was filtered with a 0.22 μm filter and stored in −80°C until ready for use.

### Enzyme-linked immunosorbent assay (ELISA) assessment of FGA

The Human FGA ELISA kit (Cloud-Clone Corp, USA) was used to quantify the secretion level of FGA in the supernatant of hEM15A shNC cells and shFGA cells following the manufacturer’s instructions.

### Flow Cytometric Apoptosis and Cell Cycle Analysis

After 24h treatment with conditioned medium of shNC or shFGA endometrial stromal cells, HUVECs were harvested. Cells apoptosis was evaluated using the Annexin-V-FITC/propidium iodide (PI) apoptosis detection kit (BD Biosciences, USA) and cell cycle assay was performed using a cell cycle assay kit (BD Biosciences, USA) according to the manufacturer’s instruction. Staining of samples was detected using the FACS Calibur flow cytometry system (BD Biosciences) and data was analyzed using Flowjo and Modfit software.

### Edu Assay

EdU assay was performed using the Cell-Light™ EdU Cell Proliferation Detection Kit (Ruibo Biotech, Guangzhou, China). In brief, cells were plated into 96-well plates (4×10^3^ cells/well), incubated with 10µM EdU for 2 h and fixed in 4% paraformaldehyde, permeabilized with 0.1% TritonX-100, incubated with 2 mg/mL glycine for 5 min. Then cells were incubated with Apollo reaction mixture for 30 min, and cell nuclei were stained with Hoechst 33342 for 30 min. Finally, the images were captured using a fluorescent microscope (Leica, Germany) and the number of positive cells were counted.

### Scratch Assay

HUVECs were seeded into 6-well plates. When 98% confluency was reached, 200μl sterile plastic pipette tips were used to scratch the cells and the medium was replaced with conditioned medium from shNC cells or shFGA cells. After 24 h, the wound healing distances were photographed and measured.

### Transwell Migration Assay

After 24h treatment with conditioned medium of shNC or shFGA endometrial stromal cells, HUVECs (3 × 10^4^/200μl) were suspended using medium without FBS. Next, cells were seeded into the upper chamber of a transwell insert (8-um pore, Corning) and 600μl of medium with 10%FBS was added to the lower chamber. After incubation for 24 hours, cells in the upper chamber were removed with a swab. The cells that migrated to the lower layer were fixed with 4% paraformaldehyde and then stained with crystal violet. Migrated cell populations were evaluated in five fields per well under a microscope.

### *In Vitro* Matrigel Tube Formation Assay

Matrigel (BD Biosciences) was thawed overnight at 4 C before use and then was added into 96-well plates 60 μL/well and solidified at 37°C for 30 min. HUVECs (2×10^4^cells/well) were resuspended with conditioned medium of shNC cells or shFGA cells and were seeded in the Matrigel-coated plate. The tube formation was observed under an inverted light microscope after 4 h incubation at 37°C. The number of meshes and tubes were calculated using Image J Angiogenesis Analyzer to assess the tube formation ability of the HUVECs.

### Immunofluorescence and Cytoskeleton Staining

After pretreated with conditioned medium for 24 h, HUVECs were seeded on coverslips cultured in 24-well plates. When the cells were attached to the coverslips, the coverslips were soaked with PBS for 3 times, 3 minutes each time. Fixed with 4% polyformaldehyde for 15 minutes, and washed with PBS for 3 minutes each time. Next, the cells were permeabilized with 0.1% Triton X-100 permeates for 10 minutes at room temperature. After being washed three times, the cells were blocked with 1% bovine serum albumin for 1 h and treated with the indicated primary antibody anti-VE-cadherin (#2500, 1:300, Cell Signaling Technology, USA rabbit, USA) at 4°C overnight, and then incubated with secondary antibodies. The cytoskeleton and nuclei were stained with 50µg/mL phalloidin (AAT Bioquest, USA) and DAPI (Solarbio, China), respectively. After being washed three times with PBS, images were acquired by a confocal microscope (Olympus FV1000, Japan).

### Immunohistochemistry Staining and Results Examination

The formalin-fixed, paraffin-embedded tissue samples were sliced into 5-μm-thick sections. The tissue slides were deparaffinized by xylene and hydrated with graded ethanol. The tissue slides were incubated with fresh prewarmed antigen retrieval buffer sodium citrate, and the buffer was heated to boiling. The tissue slides were then equilibrated to room temperature for 30 min. After being blocked by 10% goat serum for 30 min at 37°C, the tissue slides were incubated with primary antibodies anti-CD31 (ab28364, 1:100, Abcam, USA) and anti-VEGF (sc-7269, 1:50, Santa Cruz, USA) overnight at 4°C. After washing with PBS 3 times, the sections were further incubated with HRP-conjugated goat anti-rabbit IgG or goat anti-mouse secondary antibodies for 60 min at room temperature and visualized using diaminobenzidine (DAB, ZSGB-Bio) staining. The nuclei were counterstained with haematoxylin. For the negative control, nonimmune serum from rabbits or mice (1:200; ZSGB-Bio) corresponding to the primary antibodies at the respective dilutions was used. Images of five fields per slide were captured using a microscope (Leica, Germany).

The immunohistochemistry results were judged by two independent pathologists who were blinded to the clinical information. MVD was defined as the mean count of microvessels that were composed of a single or a cluster of ECs with positive CD31 staining, with a blood vessel diameter of ≤8 red blood cells, without the appearance of red blood cells and the presence of the lumen. The immunohistochemical scores of VEGF were evaluated by multiplying the percentage of positive cells (≤5% was score 0, 6%-25% was score 1, 26%-50% was score 2, ≥51% was score 3) and the staining intensity (score 0 for uncoloured, score 1 for light yellow, score 2 for brown yellow, and score 3 for brown black). Final immunohistochemical staining was defined as negative (-) for scores <1, weakly positive (+) for scores 1-3, moderately positive (++) for scores ≥3 and <6, strongly positive (+++) for scores ≥6.

### Real-Time Polymerase Chain Reaction (RT-PCR) Analysis

Total RNA from hEM15A transfected with shRNA for 48h was isolated using the TRIzol reagent ((Invitrogen, USA)). The cDNAs were synthesized by SuperScript III Reverse Transcriptase (Invitrogen) with random primers. Real-time quantitative PCR was using Power SYBR ^®^ Green PCR Master Mix (Applied Biosystems, Austin, USA) and a Bio-Rad CFX Connect Real-Time PCR System. The sequences of primers used in this study are shown in **Supplementary Table 1**. The relative expression levels of mRNA were calculated using the 2^–ΔΔCt^ method. The experiment was performed in triplicate.

### Western Blotting Analysis

Western blotting was conducted in accordance with previous work (24). The primary antibodies against FGA (ab92572, 1:2000, Abcam, USA), integrin αν (#4711, 1:1000, Cell Signaling Technology, USA), integrin β3 (#13166, 1:1000, Cell Signaling Technology, USA), AKT (#4685, 1:1000, Cell Signaling Technology, USA), P-AKT (#4060, 1:1000, Cell Signaling Technology, USA), ERK (#4695, 1:1000, Cell Signaling Technology, USA), P-ERK (#4370, 1:1000, Cell Signaling Technology, USA), FAK (#13009, 1:1000, Cell Signaling Technology, USA), P-FAK (#3282, 1:1000, Cell Signaling Technology, USA), matrix metalloproteinase-2 (MMP-2) (ab97779, 1:1000, Abcam, USA) and MMP-9 (ab137867, 1:1000, Abcam, USA) were incubated at 4°C overnight. The target proteins were visualized by the chemiluminescent gel imaging system (Bio-Rad, USA) after incubation with a secondary antibody (7074, 1:3000, Cell Signaling Technology, USA). Each experiment was tested in three replicates.

### Animals and Matrigel Plug Assay

Female BALB/c nude mice (n=18), 2–3 weeks old, were purchased from Beijing Weitong Lihua Company. The nude mice were reared in the Experimental Animal Center of Peking University People’s Hospital. The room temperature was controlled at 23 ± 2°C, the relative humidity was 45%-55%, the light time was 12 h, the mice had free access to food, and the mice were allowed to adapt for 1 week. As for Matrigel plug assays, HUVECs were treated with the conditioned medium (CM) of hEM15A shNC or shFGA cells for 24h and then harvested and suspended with 100μl CM and mixed with equal volume Matrigel. Animals were arranged into three groups and subcutaneously injected with the above prepared mixture respectively (shNC group: mixture of Matrigel and HUVECs treated with shNC cells-derived CM, shFGA group: mixture of Matrigel and HUVECs treated with shFGA cells-derived CM, shFGA+VEGF group: mixture of Matrigel and HUVECs treated with shFGA cells-derived CM and VEGF). Fourteen days after injection, the mice were humanely killed, and the plugs were gently removed using blunt dissection to avoid bleeding. The plugs were placed in paraformaldehyde for easy fixation and sectioning in the next step.

### Hematoxylin-Eosin (HE) Staining and Masson Staining

The formalin-fixed, paraffin-embedded samples from Matrigel plug assays were sliced into 5μm thick sections. HE and Masson staining of mice sections were performed using the HE staining kit and Masson Trichrome Staining Kit according to the manufacturer’s instructions (Solarbio, China). Images of per slide were captured using a microscope (Leica, Germany).

### Statistical Analysis

Statistical analysis was performed with SPSS 21.0 software. The VEGF expression between groups was analyzed by the chi-square test. The correlations between MVD level and clinical characteristics were analyzed by t test. Normal distribution of the MVD data was assessed by histograms, P-P plots, Q-Q plots and Kolmogorox-Smirnov test tests and results showed that the data of MVD in two groups met normal distribution (**Supplementary Figure S2**). Quantitative data were expressed as mean ± SEM, and the independent sample t test was used for comparison between groups. All results are statistically significant with p<0.05. All experiments were repeated at least 3 times.

## Results

### The MVD and VEGF Expression Are Increased in EM

**Figure 1:**
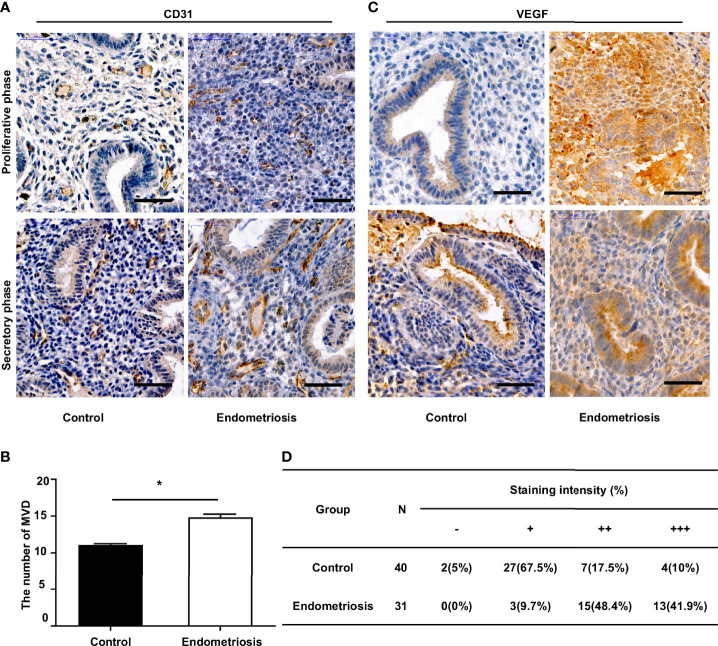
Increased MVD level and VEGF expression in the eutopic endometrium of endometriosis patients. **(A)** Representative immunohistochemical images of CD31 staining in eutopic endometrium of endometriosis patients and normal endometrium of patients without endometriosis (control), bar=50µm. **(B)** Quantitative analysis of MVD levels between endometriosis and control group. **(C)** Representative immunohistochemical images of VEGF staining in eutopic endometrium of endometriosis patients and normal endometrium of patients without endometriosis (control), bar=50µm. **(D)** Percentages of different staining intensities of VEGF in endometriosis and control group.*P < 0.05.

To assess angiogenesis activity in EM, we detected the MVD and expression of VEGF using immunohistochemistry in 31 patients with EM and 40 patients without EM as a control group. The results showed that the MVD in the eutopic endometrium of EM patients was significantly higher than that in the normal endometrium (**Figures 1A, B**). VEGF expression was moderately and strongly positive in the eutopic endometrium, which was significantly higher than that in the normal endometrium, which was mainly weakly positive (**Figures 1C, D**). We further analysed the relationship between MVD in the eutopic endometrium of EM patients and clinical characteristics and found that patients with recurrence had a higher MVD than those without recurrence, although there was no significant difference for other parameters, including age, menstrual cycle, or infertility (**Table 2** and **Supplementary Table S3**).

**Table 2:** Correlations between MVD level in eutopic endometrium and clinical characteristics of EM patients.

Clinical Characteristics	Number	MVD	*P*
Age (years)			
≤30	11	14.09±1.099	0.499
>30	20	15.00±0.778	
Menstrual cycle			
Proliferative phase	24	14.42±0.665	0.267
Secretory phase	7	15.57±1.674	
Dysmenorrhea			
Yes	22	12.27±0.706	0.479
No	9	15.67±1.323	
Infertility			
Yes	27	14.41±0.676	0.273
No	4	16.50±1.658	
Ovarian lesion			
Unilateral	20	14.25±0.804	0.369
Bilateral	11	15.45±1.012	
Peritoneal lesion			
Yes	23	14.91±0.800	0.535
No	8	14.00±0.845	
r-AFS stage			
I-II	12	15.50±1.098	0.307
III-III	19	14.16±0.758	
Recurrence			
Yes	6	18.00±0.837	0.018
No	25	14.04±0.667	

### The Expression of FGA in Endometrial Stromal Cells of EM Increases the Angiogenesis Ability of ECs *In Vitro*

**Figure 2:**
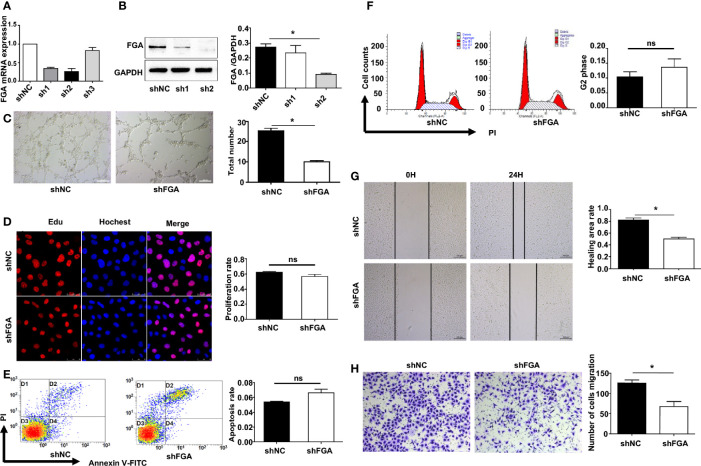
Effect of the conditioned medium (CM) of hEM15A with FGA knockdown on HUVECs. **(A, B)** The expression levels of FGA mRNA and protein were assessed by RT-PCR **(A)** and Western blot analysis **(B)** after transfection with sh1, sh2, sh3 and shNC in hEM15A, and sh2 was selected for use in the subsequent experiments as shFGA. **(C)** Tube formation assay. HUVECs were plated on Matrigel in CM of hEM15A shNC or hEM15A shFGA cells and then were observed under microscope after incubating for 4h; (left) Representative images of tube, bar=200μm. (right) Quantitative analysis of total tube number. **(D)** Edu assay showing the proliferation of HUEVCs treated with CM from hEM15A shNC and hEM15A shFGA cells for 24h. **(E, F)** Flow cytometry analysis showing that apoptosis rate **(E)** and cell cycle **(F)** of HUEVCs treated with CM from hEM15A shNC and hEM15A shFGA cells for 24h. **(G, H)** Scratch assay **(G)** and transwell assay **(H)** were conducted to assess migration ability of HUEVCs treated with CM from hEM15A shNC and hEM15A shFGA cells for 24h. * *P* < 0.05, ns: not significant, *P *≥ 0.05.

**Figure 3:**
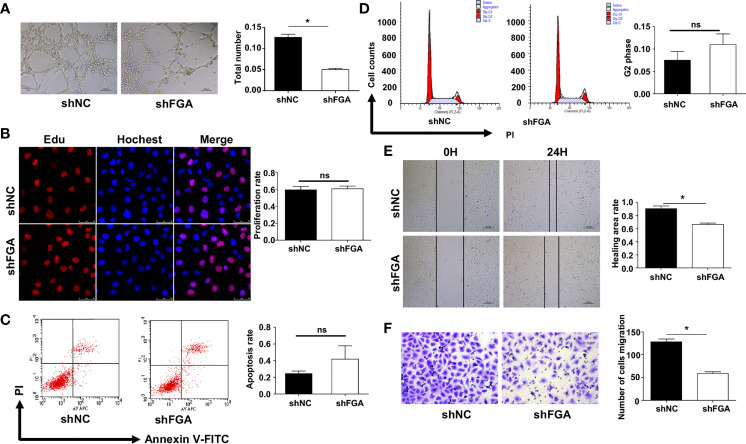
Effect of the conditioned medium (CM) of EuESCs with FGA knockdown on HUVECs. **(A)** Tube formation assay. HUVECs were plated on Matrigel in the CM of EuESCs shNC and EuESCs shFGA cells and then were observed under microscope after incubating for 4h. (left) Representative images of tube, bar=200 μm. (right) Quantitative analysis of total tube number.**(B)** Edu assay showing the proliferation of HUEVCs treated with CM from EuESCs shNC and EuESCs shFGA cells for 24h. **(C, D)** Flow cytometry analysis showing that apoptosis rate **(C)** and cell cycle **(D)** of HUEVCs treated with CM from EuESCs shNC and EuESCs shFGA cells for 24h. **(E, F)** Scratch assay **(E)** and transwell assay **(F)** were conducted to assess migration ability of HUEVCs treated with CM from EuESCs shNC and EuESCs shFGA cells for 24h. * *P* < 0.05, ns, not significant, *P  *≥ 0.05.

We next investigated whether and how FGA regulates angiogenesis in EM based on our previous studies, which demonstrated a higher level of FGA and its crucial role in disease progression in EM. First, lentiviral shRNA interference was used to knock down the expression of FGA in hEM15A cells and primary EuESCs. RT-PCR and Western blot analysis showed that sh2 could most significantly decrease the expression of FGA in endometrial stromal cells at both the mRNA and protein levels (**Figures 2A, B**), which was thus selected for use in subsequent experiments. Furthermore, ELISA results revealed the concentration of FGA in the supernatant of hEM15A shFGA was significantly lower than that of shNC cells, indicating transfection with shFGA significantly also downregulated FGA secretion in hEM15A cells (**Supplementary Table S2**). To examine the effect of FGA from endometrial stromal cells on angiogenesis, we collected the supernatant of hEM15A cells and EuESCs after FGA knockdown as conditioned medium and then treated HUVECs with them. Compared with hEM15A shNC cells, conditioned medium from hEM15A shFGA cells significantly inhibited the tube formation of HUVECs (**Figure 2C**), as well as the migration capability detected by scratch test and Transwell chamber assays (**Figures 2G, H**). However, the results from the EdU assay (**Figure 2D**) and flow cytometry (**Figures 2E, F**) showed that knocking down the expression of FGA in hEM15A cells had no significant effect on the proliferation, apoptosis or cell cycle ability of HUVECs. Moreover, the above experiments were also performed with primary EuESCs and demonstrated the same result (**Figure 3**).

### The Expression of FGA in Endometrial Stromal Cells Increases the Angiogenesis Ability of ECs *In Vivo*

**Figure 4:**
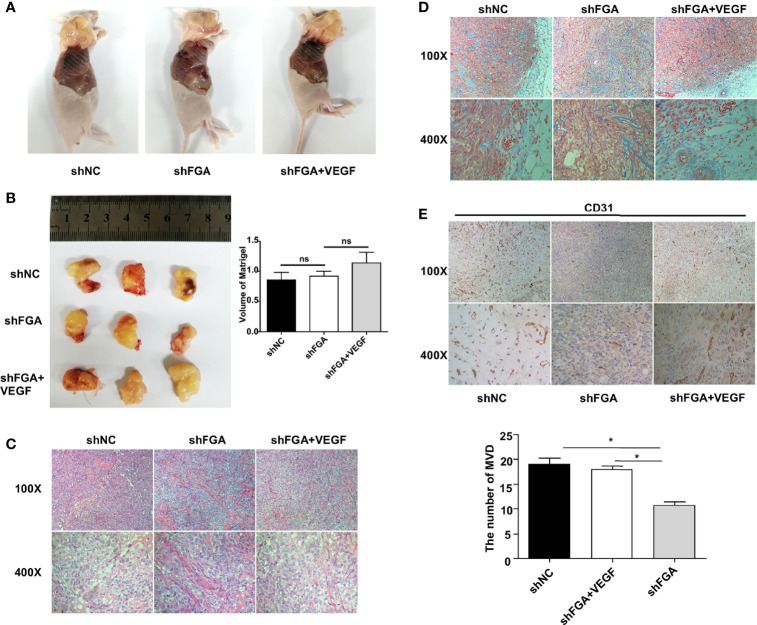
Effect of conditioned medium (CM) of hEM15A with FGA knockdown on the angiogenesis *in vivo*. **(A)** The representative anatomical images of female BALB/c nude mice after subcutaneous injection with HUEVCs treated with CM of hEM15A shNC cells (shNC group), CM of hEM15A shFGA cells (shFGA group) and CM of hEM15A shFGA cells combined with VEGF (shFGA+VEGF group). **(B)** Representative images of matrigel plugs at day14 after resection (left) and quantitative analysis of volume of plugs among three groups (right). **(C, D)** Representative images of matrigel plugs sections using HE staining **(C)** and Masson staining **(D)** showing tissue morphology, cells infiltration and angiogenesis in three groups. **(E)** Immunohistochemical images of CD31-positive endothelial cells in matrigel plugs sections (upper) and quantitative analysis (bottom) of the number of MVD in shNC group, shFGA group and shFGA+VEGF group. * *P* < 0.05, ns, not significant, *P *≥ 0.05.

To further explore the effect of FGA expression in endometrial stromal cells on the angiogenesis ability of ECs, we performed a Matrigel plug assay in nude mice. According to treatments of CM from hEM15A cell, HUVECs were divided into three groups: the shNC group, shFGA group and shFGA+VEGF group. The shFGA group showed less blood vessel growth in the plug than the shNC group, and in the shFGA+VEGF group, the Matrigel plug demonstrated neovessel formation with obvious small blood vessel-like structures, although no significant difference in plug volume was observed among the three groups (**Figures 4A, B**). In addition, HE (**Figure 4C**) and Masson staining (**Figure 4D**) showed that multiple cells surrounded by connective tissue were present in all plugs, and there were vascular-like structures in the Matrigel plugs of the shNC control group and shFGA+VEGF group, with a large number of red blood cells seen in part of the lumen; this was not observed in the shFGA group. CD31 immunohistochemical staining was further used to confirm the presence of ECs and to quantitate neovessel formation (**Figure 4E**). The results revealed a significant increase in the MVD area in the shNC control group and shFGA+VEGF group in comparison to that in the shFGA control group.

Altogether, the above data suggested the antiangiogenic ability of FGA knockdown in endometrial stromal cells *in vivo*, and this phenotype was reduced by the addition of VEGF.

### Knockdown of FGA Downregulates the Expression of Proangiogenesis-Related Factors in Endometrial Stromal Cells

**Figure 5:**
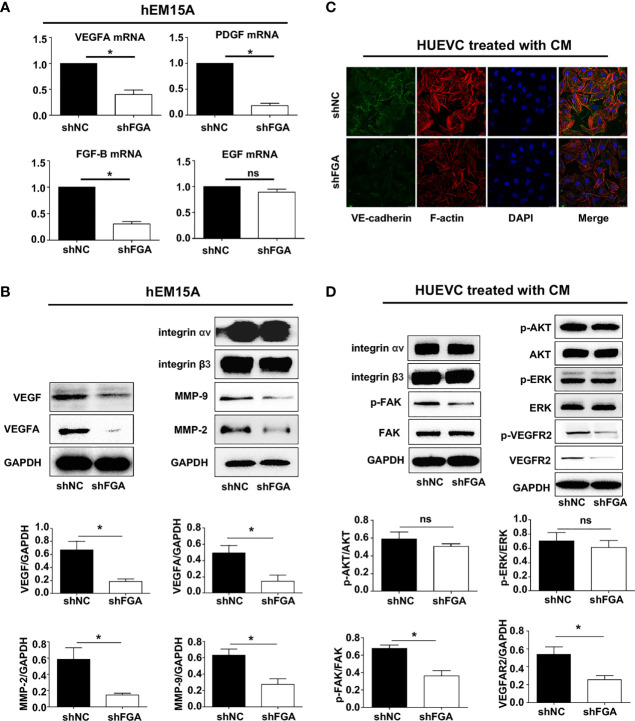
The specific mechanism of FGA on angiogenesis in endometriosis. **(A)** The relative mRNA expression of VEGFA, PDGF, FGF-B and EGF in hEM15A after FGA knockdown by shFGA compared with control group (shNC) was detected by RT-PCR. **(B)** The protein expression of VEGFA, VEGF, MMP-2 and MMP-9 in hEM15A with FGA knockdown was detected by western blotting. **(C)** Representative immunofluorescence image of HUVECs treated with CM of hEM15A shNC and hEM15A shFGA cells for 24h (green, VE-cadherin; red, F-actin; blue, DAPI-labeled nuclei). **(D)** Western blotting analysis showing protein levels of integrin αv, integrin β3 and the signalling pathway of VEGFR2, FAK, AKT, ERK in HUVECs treated with CM of hEM15A shNC and hEM15A shFGA cells for 24h. * *P* < 0.05, ns, not significant, *P *≥ 0.05.

After confirming the proangiogenic role of FGA, we proceeded to investigate the underlying molecular mechanism by which FGA modulates the angiogenesis of ECs. Many studies have reported that endometrial stromal cells in EM possess strong paracrine and autocrine activity, and thus, we first evaluated the influence of FGA on the expression of angiogenesis-related factors in hEM15A using RT-PCR and Western blotting. The results showed that knockdown of FGA expression significantly decreased the mRNA levels of VEGFA, PDGF and FGF-B (**Figure 5A**) and the protein levels of VEGFA, VEGF, MMP-2 and MMP-9 (**Figure 5B**) in hEM15A cells.

### FGA Knockdown in Endometrial Stromal Cells Inhibits Endothelial Cytoskeleton Formation, VE-Cadherin Expression and the VEGFR2 and FAK Signalling Pathways in HUVECs

Next, we sought to assess the molecular changes in HUVECs after treatment with conditioned medium from hEM15A cells with FGA knockdown. Immunofluorescence analysis showed that compared with conditioned medium from hEM15A shNC cells, conditioned medium from shFGA hEM15A cells significantly reduced the abundance of microfilaments and pseudopodia (as shown by F-actin staining) and caused an obvious decrease in VE-cadherin expression in ECs (**Figure 5C**). Furthermore, we examined the activity of the downstream FAK, AKT, ERK and VEGFR signalling pathways in HUVECs by Western blotting. The results revealed that the expression of P-FAK, P-VEGFR2 and VEGFR2 was downregulated after treatment of hEM15A shFGA cells-derived conditioned medium, but the expression of integrin αv, β3, FAK, AKT, P-AKT, ERK and P-ERK and did not change significantly (**Figure 5D**). The results suggested that activating VEGFR2 and the FAK signalling pathway may be involved in promoting the angiogenesis process of FGA.

## Discussion

**Figure 6:**
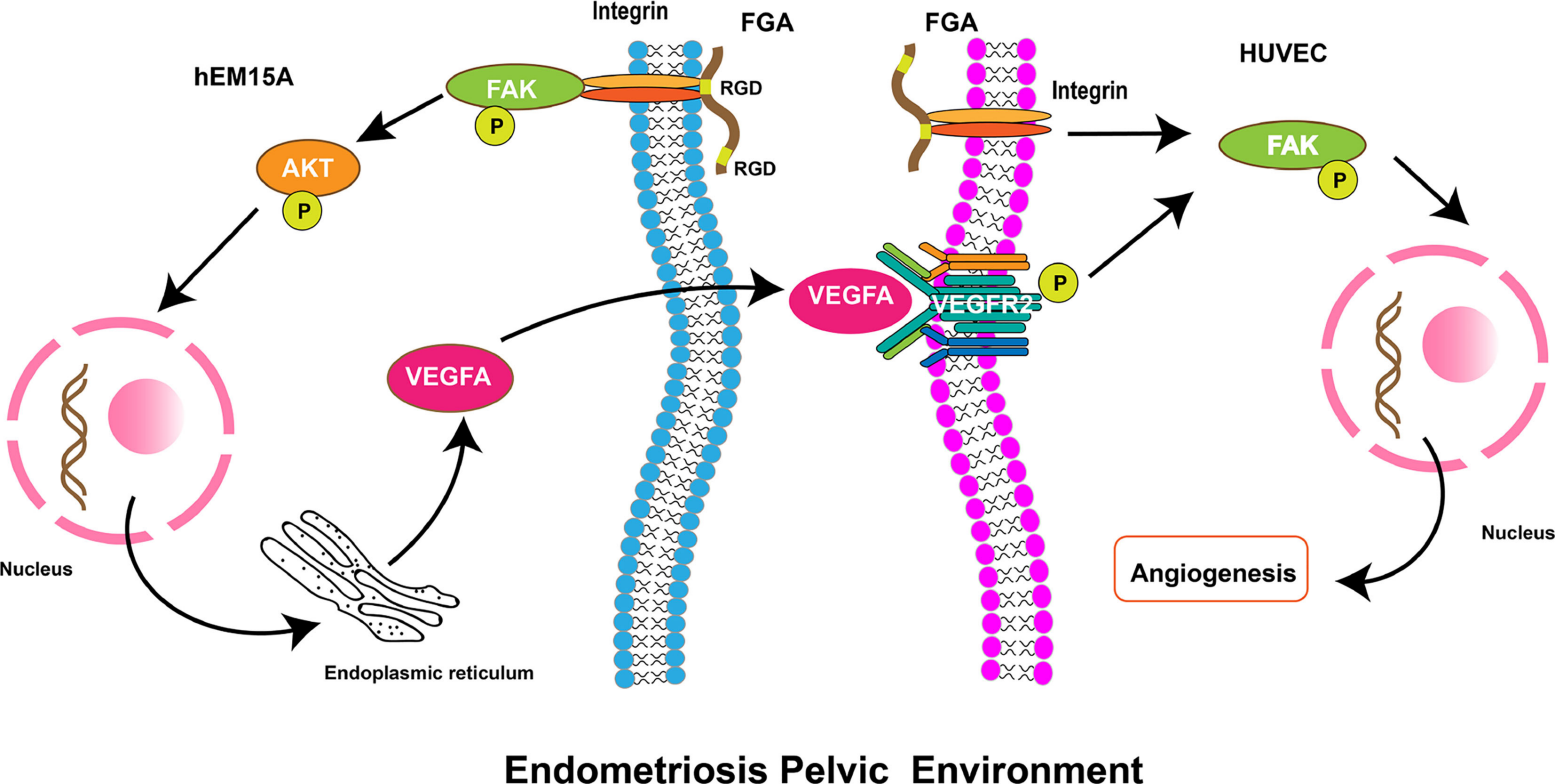
Schematic diagram showing the role of FGA on angiogenesis in endometriosis. FGA in endometrial cells could activate the FAK-AKT signalling pathway by binding integrin *via* the RGD region and increase the expression of VEGFA. VEGFA from endometrial cells binds and phosphorylates its receptor, VEGFR, on endothelial cells and then activates the downstream FAK pathway to enhance angiogenesis.

Previous studies have indicated that FGA could promote adhesion and invasion in EM and is associated with the stage of EM. However, it remains unknown whether endometrial stroma cells-derived FGA can affect endometriotic lesion growth by promoting angiogenic processes. In this study, we showed that FGA is associated with MVD and VEGF in eutopic endometrial tissue from EM patients, which indicated that FGA may play a role in the angiogenic processes of EM. In addition, FGA knockdown in endometrial stromal cells significantly inhibited the angiogenesis and migration of HUVECs and the expression of angiogenic factors, such as VEGF, PDGF and FGF-B. Further analyses showed that downregulating the expression level of FGA may also attenuate angiogenesis *in vivo*. Our results showed that FGA may activate the VEGFA-VEGFR2-FAK signalling pathway in ECs to promote angiogenesis (**Figure 6**). Taken together, these data provide the first pieces of evidence regarding the role of FGA in EM angiogenesis and the underlying mechanisms responsible for FGA-associated angiogenesis.

Considering Sampson’s implantation theory, EM is the result of shed endometrial tissues implantation on the peritoneal surface after retrograde menstruation (25). A new blood supply is recognized as an essential process in the growth and development of the endometriotic lesions. Moreover, the activation of angiogenesis appears to be a key factor in the pathogenesis of EM. As reported, transplanted human endometrial tissue fragments have been validated as endometriotic lesions, and this is a common approach to establish an experimental model of EM (26, 27). Studies have reported that shed endometrial tissues exhibit physiological characteristics, including morphological and biochemical characteristics, and are equipped with fully functional microvascular networks, which are crucial for the ectopic implantation of the human endometrium. According to this hypothesis, early endometriotic lesions are characterized by increased MVD. Many studies found a significantly increase in MVD in the eutopic endometrium from adenomyosis patients compared with the control endometrium (28, 29). Ota H et al. also found that the eutopic endometrium in adenomyosis exhibited an increase in the mean and total surface area of capillaries, irrespective of the menstrual cycle phase, compared with the control endometrium (30). However, there has been little research on MVD in the eutopic endometrium from EM patients compared with the normal endometrium in patients without EM. In this study, the expression of MVD in the eutopic endometrium was further confirmed by immunohistochemistry. Our study indicated that the level of MVD was significantly higher in EM patients than in healthy controls, irrespective of the menstrual cycle phase. Our study previously indicated that the expression of FGA in the eutopic endometrium was significantly increased compared with that in the normal endometrium and was positively associated with advanced clinical stages of EM and a higher recurrence rate (14). Similarly, this current research also demonstrated a positive relationship between MVD and EM recurrence. Thus, we hypothesized that overexpression of FGA in the eutopic endometrium of patients might have a crucial role in endometriotic angiogenesis.

Angiogenesis is an essential process involving the formation of new blood vessels and is tightly regulated by a plethora of factors, including interactions between the cellular matrix, proteolytic enzymes, and proangiogenic factors. The most important cell type activated during angiogenesis is ECs (31). The angiogenic process is largely accomplished by coordinated actions of ECs, such as cell proliferation, differentiation, apoptosis, and guided migration (32, 33). Thus, the appropriate regulation of EC biological functions affects the angiogenic process. It is largely believed that angiogenesis is a main feature of EM, but the underlying mechanisms of angiogenesis in EM have not been completely elucidated (34). Our data suggested that FGA was associated with angiogenesis with a positive relation to MVD at the histological level. However, there is no information available concerning the role of FGA in the regulation of endothelial cell function. In this study, knockdown of FGA by using a shRNA in hEM15A and EuESCs directly resulted in a decrease in HUVEC cell migration and tube formation ability *in vitro*. Subcutaneous injection of HUVECs pretreated with supernatants of hEM15A by shFGA in node mice showed weaker angiogenic capacity compared with shNC control group, while the addition of VEGF could alleviate the inhibitory effect of shFGA cells. Moreover, the study also found that downregulated FGA attenuated EC proliferation and increased apoptosis, but no significant statistics were shown, which indicated that FGA may play a critical role in angiogenesis by promoting the migration ability of ECs but not proliferation and apoptosis abilities.

Angiogenesis is determined by not only the degradation of the basement membrane from existing blood vessels but also the proliferation, migration and tube formation of ECs influenced by growth factors (17, 35). Any change in these stages will influence angiogenesis. Moreover, several studies have reported that angiogenesis in tumours is involved in ECs and the crosstalk between ECs and other cell types in the tumour microenvironment (36–38). Although EM is a benign disease, it exhibits many features similar to malignancy. Canosa et al. found that human endometrial mesenchymal stromal cells (E-MSCs) isolated from eutopic and ectopic endometrial tissue have the potential to differentiate into ECs and are possibly involved in support of angiogenesis (39). Yerlikaya et al. analysed the expression of angiogenic factors in women with EM and without EM. The results showed that TGFB2, FGF and VEGFR were significantly different between eutopic and control endometrium, which implied that angiogenic factors in eutopic endometrium may play a role in the pathogenesis of EM. VEGF, PDGF, FGF and EGF have been reported to mediate angiogenesis, including endothelial cell behaviours (40–42). In the present study, our data indicated that FGA knockdown reduced the expression of VEGF, PDGF and FGF in endometrial stromal cells at the mRNA and protein levels. The results indicated that the angiogenic factors released from endometrial stromal cells may be influenced by the expression of FGA, which suggested that FGA promotes EM angiogenesis by increasing the expression of various proangiogenic factors, including VEGF, PDGF and FGF. The initial phase of angiogenesis is an event in which ECs exit existing blood vessels by ECM breakdown. The ECM balance may be affected by MMPs and tissue inhibitors of metalloproteinases (TIMPs). Matrix metalloproteinases (MMPs) degrade ECM components as members of a zinc-dependent endopeptidase family (43). The major MMPs involved in the process of angiogenesis are MMP-2 and MMP-9. Abnormal expression of MMP-2 and MMP-9 is associated with multiple stages of tumour growth, vascular invasion, tumour progression, and metastasis (44). In this study, we found that knockdown of FGA by using shRNA in hEM15A directly decreased the expression of MMP-2 and MMP-9. Taken together, the above results may indicate FGA’s involvement in the angiogenic process of EM through regulating the expression of angiogenic factors VEGF, PDGF, FGF, MMP-2 and MMP-9.

Angiogenesis is stimulated through a number of molecules and signalling pathways that are dependent on the behaviour of ECs. Among the proangiogenic factors, VEGF has been indicated to be one of the most important angiogenesis growth factors involved in normal and pathological angiogenic processes. The VEGF family contains several members, such as VEGF-A, VEGF-B, VEGF-C, VEGF-D, VEGF-E and placental growth factors. VEGFA, as the most functional form of VEGF, binds to its predominant receptor VEGFR2 expressed on vascular ECs to exert its proangiogenic activity (45–47). According to our results, FGA knockdown reduced VEGFA expression in hEM15A, and the supernatant of hEM15A cells with FGA knockdown decreased the phosphorylation of FAK and VEGFR2 in HUVECs, which suggests that FGA may promote EM angiogenesis by regulating the expression of angiogenic factors in endometrial stromal cells and activating the VEGFR2 and FAK signalling pathways in ECs. Despite the above promising findings, there are also certain limitations to our research. First, the strong relationship between the expression of FGA and VEGF in women with EM could be clarified in the same cohort, although they both showed increased levels in EM. Second, further investigation of the effect of FGA on angiogenesis could also be explored using animal models of EM to enhance our research results. Third, based on our previous finding that RGD antagonist treatment could attenuate the promotion of FGA on the migration and invasion ability of endometrial stromal cells (24), many future experiments could be conducted to investigate whether and how the RGD region of FGA is involved in angiogenesis, therefore providing new drug choices for EM patients.

In conclusion, this study indicated that the increased vascular activity of the eutopic endometrium in EM patients is closely related to the high expression of FGA. FGA may activate the VEGFA-VEGFR2-FAK signalling pathway in ECs to promote angiogenesis by regulating the expression of VEGFA, VEGF and MMPs in endometrial stromal cells of EM. This not only provides evidence for the involvement of FGA in angiogenesis but also provides a new idea for antiangiogenic therapy for EM patients.

## Data Availability Statement

The raw data supporting the conclusions of this article will be made available by the authors, without undue reservation.

## Ethics Statement

The studies involving human participants were reviewed and approved by the Ethics Committee of Peking University People’s Hospital. The patients/participants provided their written informed consent to participate in this study. The animal study was reviewed and approved by the Animal Experiment Ethical Committee of Peking University People’s Hospital. Written informed consent was obtained from the individual(s) for the publication of any potentially identifiable images or data included in this article.

## Author Contributions

HL and EC - study design, experimentation, data analysis and interpretation, figure preparation, and writing and revising of manuscript. HC and XY - involvement in isolation and culture of human eutopic endometrial cells described in this study. RM - data acquisition and analysis. HZ - study design, scientific guidance, and collection of clinical samples. XC - obtainment of funding, ethics approval for the study, and scientific guidance and revising of manuscript. All authors contributed to the article and approved the submitted version.

## Funding

This study was supported by the National Natural Science Foundation of China (grant no. 81971360 and no. 81671431) and the Beijing Municipal Natural Science Foundation (grant no. 7222206).

## Conflict of Interest 

The authors declare that the research was conducted in the absence of any commercial or financial relationships that could be construed as a potential conflict of interest.

## Publisher’s Note

All claims expressed in this article are solely those of the authors and do not necessarily represent those of their affiliated organizations, or those of the publisher, the editors and the reviewers. Any product that may be evaluated in this article, or claim that may be made by its manufacturer, is not guaranteed or endorsed by the publisher.
