# Supplementary material for: FGA Controls VEGFA Secretion to Promote Angiogenesis by Activating the VEGFR2-FAK Signalling Pathway
Source: Front Endocrinol (Lausanne). 2022 Apr 13;13:791860. doi: 10.3389/fendo.2022.791860 (PMC9043601; doi:10.3389/fendo.2022.791860)
Supplement: Supplementary file 1 [file DataSheet_1.docx]

Supplementary Material

1. **Supplementary Tables**

**Supplementary Table S1.** Summary of primers used in this study.

| **Gene** |  | **Primer sequences (5’ - 3’)** |
| --- | --- | --- |
| FGA | Forward | GAAGACTGGAACTACAAATGCC |
|  | Reverse | GTACCTTCTGAAGCTGGCTC |
| VEGF-A | Forward | GAGCCTTGCCTTGCTGCTCTAC |
|  | Reverse | CACCAGGGTCTCGATTGGATG |
| PDGF | Forward | GATCCGCTCCTTTGATGATCTC |
|  | Reverse | GGTCATGTTCAGGTCCAACTC |
| EGF | Forward | GAAGCATTGGACAAGTATGCAT |
|  | Reverse | CAGCTTCTGAGTCCTGTAGTAG |
| FGF-B | Forward | CATCAAGCTACAACTTCAAGCA |
|  | Reverse | CCGTAACACATTTAGAAGCCAG |
| GAPDH | Forward | TGCACCACCAACTGCTTAGC |
|  | Reverse | GGCATGGACTGTGGTCATGAG |

**Supplementary Table S2.** The secretion of FGA in the supernatant of hEM15A cells transfected with shFGA and shNC

| Group | Concentration（ng/ml） | *P* |
| --- | --- | --- |
| shNC | 32.00±1.26 | <0.05 |
| shFGA | 10.64±1.12 |  |

**Supplementary Table S3.** The subgroup analysis of MVD

| Group | Subgroup | MVD (mean±SEM) | *P* |
| --- | --- | --- | --- |
| All patients  (n=71) | Proliferative phase (n=43) | 13.03±0.50 | 0.132 |
|  | Secretory phase (n=28) | 11.74±0.73 |  |
| All patients  (n=71) | Control  (n=40) | 10.85±0.40 | **<0.0001** |
|  | Endometriosis  (n=31) | 14.68±0.63 |  |
| Proliferative phase  (n=43) | Control  (n=19) | 11.28±0.53 | **0.001** |
|  | Endometriosis  (n=24) | 14.42±0.66 |  |
| Secretory phase  (n=30) | Control  (n=21) | 10.46±0.58 | **0.001** |
|  | Endometriosis  (n=7) | 15.57±1.67 |  |
| Control  (n=40) | Proliferative phase  (n=19) | 11.28±0.53 | 0.309 |
|  | Secretory phase  (n=21) | 10.46±0.58 |  |
| Endometriosis  (n=31) | Proliferative phase  (n=24) | 14.42±0.66 | 0.453 |
|  | Secretory phase  (n=7) | 15.57±1.67 |  |

1. **Supplementary Figures**

**Supplementary Figure S1.** The flow chart of this study.

**Supplementary Figure S2.** Normality test of MVD in Control Group and EM Group. (A-B): The histogram of MVD in Control Group (A) and EM Group (B). (C-D): Normal P-P plot of MVD in Control Group (C) and EM Group (D). (E-F) Normal Q-Q plot of MVD in Control Group (E) and EM Group (F). (G-H) The results of Kolmogorov-Smirnov Test for MVD in Control Group (G) and EM Group (H). All results showed that the data of MVD in two groups met normal distribution.
